# Analytical and clinical evaluation of the light‐initiated chemiluminescent assays for measurement of human thyroid hormones

**DOI:** 10.1002/jcla.24266

**Published:** 2022-03-21

**Authors:** Jing Li, Youyuan Huang, Guiming Xiang, Enjun Xu, Tao Chen, Ming Yang, Jie Zhang

**Affiliations:** ^1^ 594822 Clinical Laboratory Peking University International Hospital Beijing China; ^2^ 26447 Department of Endocrinology Peking University First Hospital Beijing China; ^3^ Clinical Laboratory The Second Affiliated Hospital of Army Medical University Chongqing China; ^4^ 36639 Department of Clinical Laboratory First Affiliated Hospital of Anhui Medical University Hefei China

**Keywords:** LiCA 800 analyzer, light‐initiated chemiluminescent assay, performance evaluation, thyroid assay, thyroid disease

## Abstract

**Background:**

Light‐initiated chemiluminescent assay (LiCA) is a new homogeneous immunoassay. The aim of this study was to evaluate the analytical and clinical performance of the assays for the detection of thyroid hormones based on the fully automated LiCA 800 analyzer.

**Methods:**

Analytical validations of the LiCA thyroid assays (TSH, FT3, FT4, T3, and T4) included precision, linearity, analytical sensitivity, interference, and method comparison applying the protocols of the Clinical and Laboratory Standards Institute (CLSI). The diagnostic performance was assessed by the receiver operating characteristic (ROC) curve analysis with different assay schemes for the diagnosis of hyperthyroidism and hypothyroidism.

**Results:**

Within‐run and within‐lab precisions (%CV) of the five assays ranged from 1.06 to 6.40% at all concentrations evaluated. A satisfactory linearity was verified over the entire measuring range for TSH, T3, and T4 (*R* > 0.99, change in recovery <10%, *p *= 0.000 all). Paired‐comparison measurements presented a comparable assay for each of the five assays (*R* > 0.96, median bias <5%, *p *< 0.0001 all) between LiCA and Cobas across three institutes. The diagnostic accuracy of the LiCA assays for hyperthyroidism or hypothyroidism was quantified by the areas under curves (AUC) as 0.925 or 0.832 with the five‐assay panel (TSH, FT3, FT4, T3, and T4) and as 0.921 or 0.811 with the three‐assay panel (TSH, FT3, and FT4), respectively. No significant difference was found between the AUC of LiCA and that of DxI, Cobas, or Centaur (*p *> 0.3 all).

**Conclusion:**

LiCA 800 provides a precise and high‐throughput immunoassay platform for detection of thyroid hormones. It is acceptable for clinical use.

## Graphical abstract

In this study, we have validated the analytical and diagnostic performance of the LiCA thyroid assays. Paired‐comparison measurements demonstrated a reasonable concordance for each of the assays (*R* > 0.96, median bias <5%, *p *< 0.0001 all) between LiCA and Cobas across three institutes. To further characterize the clinical performance of the LiCA assays for the diagnosis of hyperthyroidism and hypothyroidism, we compared the AUCs with the five‐assay panel (TSH, FT3, FT4, T3, and T4) and with the three‐assay panel (TSH, FT3, and FT4) between LiCA and other three commonly used methods (DxI, Cobas, and Centaur). No significant difference was found (*p* > 0.3 all), and this confirmed the diagnostic accuracy and applicable in clinical practice of the LiCA thyroid assays.

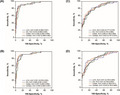

## 1 INTRODUCTION

Laboratory tests of thyroid hormones are essential for clinical diagnosis and patient management of thyroid diseases.^1^, ^2^ The key assays include thyroid‐stimulating hormone (TSH), free triiodothyronine (FT3), free thyroxine (FT4), triiodothyronine (T3), and thyroxine (T4). With overt advantages on high sensitivity and specificity, full automation, high‐throughput, and non‐radioactive contamination, chemiluminescent immunoassay (CLIA) is gradually taking the leading position for the thyroid tests in clinical laboratory.^3^ Due to lack of harmonization on the international level of reference materials, detection of thyroid assays could be variant from different platforms based on different methodologies. Thereby, a performance validation is necessary before a new assay could be admitted in clinical application.

In this study, we introduced a new homogeneous light‐initiated chemiluminescent assay (LiCA^®^) that was derived from the luminescent oxygen‐channeling immuno‐technology described by Ullman et al.^4^, ^5^ The key analytical characteristics of the five thyroid assays (TSH, FT3, FT4, T3, and T4) were assessed following the guidelines of the Clinical and Laboratory Standards Institute (CLSI). In addition, the diagnostic performance was analyzed by the receiver operating characteristic (ROC) curve with different assay schemes for the diagnosis of hyperthyroidism and hypothyroidism.

## 2 MATERIALS AND METHODS

### 2.1 Study samples

The evaluation study of the five thyroid assays (TSH, FT3, FT4, T3, and T4) was conducted at the clinical laboratory of Peking University international Hospital. Additional method comparison between LiCA and Cobas was validated at other two institutes in China. All samples were collected from residual and de‐identified patient sera within 2 h after clinical routine thyroid assays. We selected specimens based on serum quality, volume, and analyte concentrations. Subjects with visible icterus, lipemia, or hemolysis were excluded. Unless otherwise noted, specimens were stored at −20°C for no longer than 30 days with one freeze‐thaw cycle in this study.

### 2.2 LiCA^®^ thyroid assays

The LiCA^®^ thyroid assay (Chemclin Diagnostics, Beijing, China) uses two nanobeads (sensitizer and emission) to bridge a fully homogeneous immunoassay. When the antigen‐antibody complex is formed after incubation, the distance between two beads is less than 200 nm. Singlet oxygen generated from the sensitizer by light at 680 nm diffuses to the emission bead across the complex, thus triggering a chemiluminescent reaction. In contrast, no emission occurs if there is no antigen‐antibody reaction, as a longer distance (>200 nm) blocks energy transmission. Therefore, no‐wash is necessary to separate the immune complex from free components in the cuvette, and thus, this test delivers a fast and stable measurement.^6^, ^7^, ^8^ The LiCA 800 analyzer used in this study is a fully automated, random‐access, and high‐throughput (600 tests per hour) immunoassay platform. Time to the first report for each of the LiCA thyroid assays (TSH, FT3, FT4, T3, and T4) is 32 min. The assay specifications have been summarized in Table 1.

**TABLE 1:** Specifications of the LiCA thyroid assays

Assay	Sample volume	Measuring range	Reference range	Traceability	Antibodies used	Immunoassay principle
TSH	25 μl	0.010–100 mIU/L	0.270–4.200 mIU/L	WHO IRP 80/558	Mouse monoclonal	Sandwich
FT3	10 µl	1.54–76 pmol/L	3.10–6.80 pmol/L	Manufacturer reference	Sheep monoclonal	Competitive
FT4	10 µl	3.86–75 pmol/L	12.00–22.00 pmol/L	Manufacturer reference	Mouse monoclonal	Competitive
T3	10 µl	0.62–9.22 nmol/L	1.30–3.10 nmol/L	USP grade material	Sheep monoclonal	Competitive
T4	10 µl	12.87–410 nmol/L	66.00–181.00 nmol/L	USP grade material	Mouse monoclonal	Competitive

Abbreviations: IRP, International Reference Preparation; USP, United States Pharmacopeia; WHO, World Health Organization.

### 2.3 Precision study

A precision study was performed for each of the LiCA thyroid assays in accordance with the CLSI EP15‐A3 protocol.^9^ Repeatability (within‐run) and within‐laboratory (intermediate) imprecision (coefficient of variation, CV) were determined using three levels of pooled human sera. 25 measurements were collected for each sample in 5 runs over 5 days.

### 2.4 Linearity study

The linearity study (for TSH, T3, and T4) followed the CLSI EP6‐A protocol.^10^ Two human serum samples for each of the assays were collected according to the analytical upper and lower limits of the manufacturer's claims. Eleven dilution pools were prepared for linearity experiments. Each dilution was measured 4 times, and linearity was evaluated by regression analysis.

### 2.5 Analytical sensitivity study

We followed the CLSI EP17‐A2 protocol ^11^ to examine the limit of blank (LoB), limit of detection (LoD), and limit of quantitation (LoQ) for the LiCA thyroid assays. The LoB experiment was performed with the zero‐point manufacturer's calibrator. LoD and LoQ were determined using serial low levels of pooled sera. Each sample was aliquoted into 20 tubes. Triplicate measurements were performed during 20 runs over 5 days (*n* = 60).

### 2.6 Interference study

To evaluate for possible interference of hemolysis, lipemia, and icterus in the LiCA thyroid assays, three levels of patient serum samples were spiked according to manufacturer's claims with interferents that included hemoglobin (2.5 g/L), triglycerides (5.65 mmol/L), and bilirubin (171 μmol/L), respectively. Each sample was measured in duplicate, and percent recovery was calculated for the evaluation of interference.

### 2.7 Method comparison study

A three‐site comparison study was carried out for the LiCA and Cobas thyroid assays following the guideline of CLSI EP9‐A2.^12^ Serum specimens were collected from randomly selected patients with analyte concentrations across the analytical measuring range. All samples were assayed on the same day with the Cobas e602 (Roche Diagnostics) and LiCA 800 analyzers. Measuring agreement was evaluated by linear regression and bias plot.

### 2.8 Clinical performance study

A total of 377 serum specimens were enrolled from patients with suspected thyroid dysfunction at presentation, of which 55 subjects were finally confirmed with hyperthyroidism and 62 with hypothyroidism. The diagnosis was made in the presence of hyperthyroidism or hypothyroidism‐related signs and symptoms and laboratory testing support by independent clinicians. Results of the Centaur‐thyroid‐assays (Siemens Healthcare Diagnostics) were used for adjudication of the final diagnosis. Patients who had been receiving treatment for thyroid diseases were excluded. Each sample was separated into four tubes and stored at −80°C. Measurements were recorded by Cobas e602, DxI 800 (Beckman Coulter), Centaur XP, and LiCA 800 on the same day. Diagnostic performance was analyzed by receiver operating characteristic (ROC) curve.

### 2.9 Statistical analysis

Statistical analysis was performed with the software program SPSS (IBM Corp.), MedCalc (MedCalc Software Ltd.) and Excel (Microsoft Corp.). Concordance of paired measurements was assessed by Passing–Bablok linear regression and Bland–Altman bias plot. ROC curves were computed with five‐assay panel (TSH, FT3, FT4, T3, and T4) and three‐assay panel (TSH, FT3, and FT4) for two groups of patients (hyperthyroidism and hypothyroidism), respectively. Areas under curves (AUCs) were calculated to quantify the diagnostic accuracy of the assays as described by DeLong et al.^13^ A statistical significance was considered as *p *< 0.05.

## 3 RESULTS

### 3.1 Precision

As shown in Table 2, the within‐run CV% of each assay ranged from 1.06% to 5.63% and the within‐lab CV% from 1.55% to 6.40%, across low, middle, and high levels of pooled human sera. Our findings revealed that LiCA provided a good precision in thyroid assays.

**TABLE 2:** Precision analysis with the EP15‐A3 protocol

Assay	Mean (*n* = 25)	Within‐run precision		Within‐lab precision	
		SD	%CV	SD	%CV
TSH (mIU/L)	0.065	0.002	3.65	0.003	4.24
	1.663	0.034	2.06	0.041	2.46
	22.941	0.407	1.78	0.580	2.53
FT3 (pmol/L)	1.65	0.079	4.79	0.088	5.34
	5.25	0.264	5.03	0.279	5.32
	14.04	0.791	5.63	0.898	6.40
FT4 (pmol/L)	6.55	0.135	2.07	0.172	2.63
	15.79	0.499	3.16	0.515	3.26
	34.93	1.347	3.86	1.477	4.23
T3 (nmol/L)	0.65	0.007	1.06	0.011	1.67
	1.99	0.037	1.87	0.051	2.57
	6.49	0.201	3.08	0.193	2.97
T4 (nmol/L)	41.37	0.543	1.31	0.641	1.55
	127.83	2.129	1.67	2.501	1.96
	273.49	9.742	3.56	10.431	3.81

Abbreviations: CV, coefficient of variation; SD: standard deviation.

### 3.2 Linearity

The regression equation and Pearson's correlation coefficient between the expected and measured values were (Y = 1.024X − 1.663, *R* = 0.999) for TSH, (Y = 0.966X + 0.040, *R* = 0.997) for T3, and (Y = 0.973X − 1.450, *R* = 0.998) for T4, respectively. *p *= 0.000 all. The percent recovery obtained for each dilution was 96.4%–106.2% for TSH, 94.1%–107.4% for T3, and 95.6%–107.8% for T4, respectively. The hypothesis of a linear fit was accepted for each of the assays.

### 3.3 Analytical sensitivity

The results of LoB, LoD, and LoQ (20% CV) for the LiCA thyroid assays were displayed in Table 3. Our observations agreed with the manufacturer's claims.

**TABLE 3:** Determination of analytical sensitivity with the EP17‐A2 protocol

Assay	Observed values			Manufacturer claimed Detection limit
	LoB	LoD	LoQ	
TSH (mIU/L)	0.001	0.002	0.002	≤0.010
FT3 (pmol/L)	0.20	0.34	0.72	≤1.54
FT4 (pmol/L)	1.00	1.28	1.28	≤3.86
T3 (nmol/L)	0.11	0.17	0.22	≤0.62
T4 (nmol/L)	2.04	6.10	9.40	≤12.87

Abbreviations: LoB, limit of blank; LoD, limit of detection; LoQ, limit of quantitation (at 20% coefficient of variation).

### 3.4 Interferences

No analytical interference was observed for the LiCA thyroid assays in the presence of hemoglobin (2.5 g/L), triglycerides (5.65 mmol/L), and bilirubin (171 μmol/L) as claimed by the manufacturer (Table 4). The percent recovery for each of the assays was determined to be between 92.5% and 108.0% across low, middle, and high levels of serum samples.

**TABLE 4:** Interference study for endogenous substances

Assay	Baseline concentration	Percent recovery after spiking the interferents		
		Hemoglobin (2.5 g/L)	Triglycerides (5.65 mmol/L)	Bilirubin (171 µmol/L)
TSH	0.237	96.7%	92.5%	97.0%
(mIU/L)	2.871	93.9%	96.3%	94.0%
	23.146	92.7%	101.3%	94.0%
FT3	1.88	106.3%	94.6%	107.2%
(pmol/L)	4.96	104.1%	94.4%	106.1%
	12.95	97.4%	95.0%	103.2%
FT4	6.09	102.8%	95.9%	103.2%
(pmol/L)	18.17	107.7%	98.4%	104.4%
	40.42	103.6%	93.4%	105.9%
T3	0.89	105.7%	98.5%	108.0%
(nmol/L)	2.22	103.3%	98.8%	107.6%
	5.27	101.9%	105.1%	101.9%
T4	38.24	98.5%	96.4%	107.0%
(nmol/L)	116.25	98.8%	94.8%	106.3%
	220.23	104.6%	99.9%	107.6%

### 3.5 Method comparison

**FIGURE 1:**
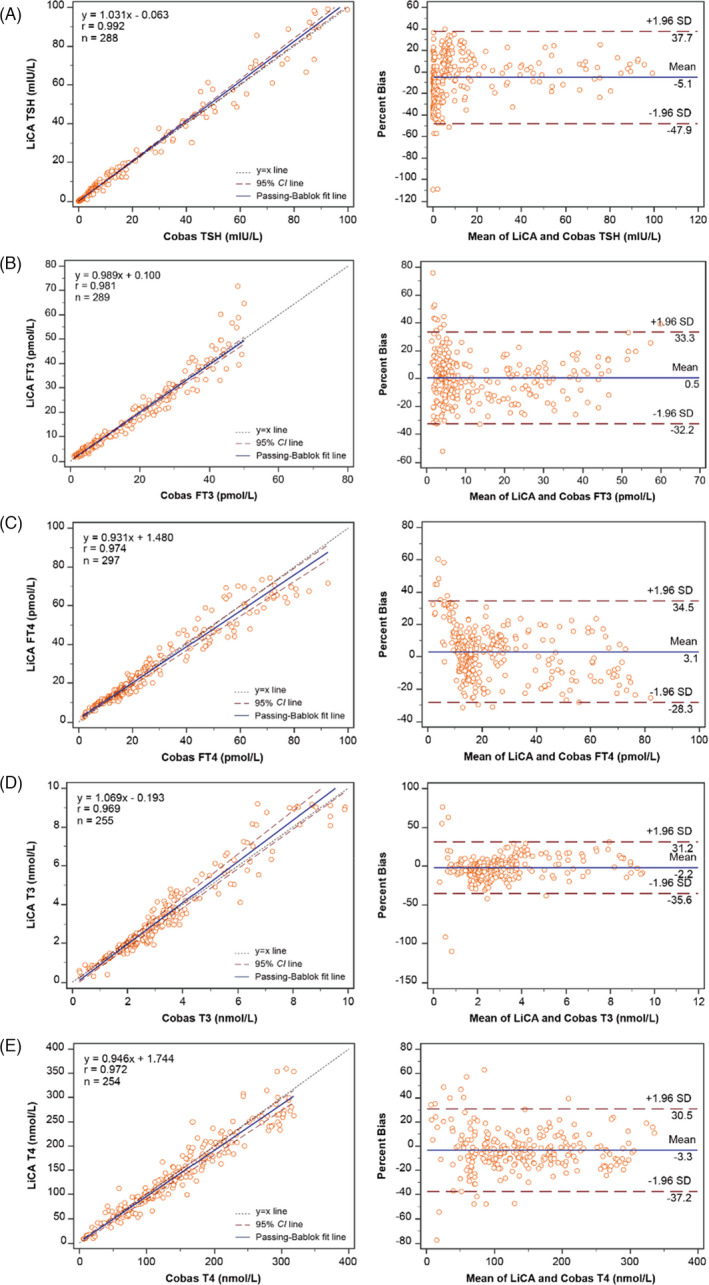
Passing‐Bablok linear regression and Bland–Altman plot analyses for measurement comparison between LiCA and Cobas thyroid assays in serum specimens using the EP9‐A2 protocol. Abbreviations: N, sample number; R, Spearman's rank correlation coefficient; SD, standard deviation

A method comparison study between LiCA and Cobas was performed using a larger pool of serum specimens across the analytical measuring range in our lab. Figure 1(A–E) showed the paired plots of Passing‐Bablok regression and Bland–Altman bias for each of the thyroid assays (TSH, FT3, FT4, T3, and T4). The Spearman's correlation coefficient *R* (95% CI: confidence interval) was 0.992 (0.990–0.994), 0.981 (0.976–0.985), 0.974 (0.967–0.979), 0.969 (0.961–0.976), and 0.972 (0.964–0.978), respectively (*p *< 0.0001 all). The median percent difference (95% CI) was −3.55% (−7.01% ~ −1.10%), 0.40% (−2.07% ~ 2.17%), 2.90% (0.33% ~ 5.69%), −3.00% (−4.56% ~ 0.01%), and −3.95% (−6.20% ~ −2.20%), respectively. A paired‐comparison experiment was repeated in other two institutes in China, and results reproduced the comparable measurements between the LiCA and Cobas thyroid assays. The detailed analyses of the multicenter comparison were summarized in Table 5 as a quick overview.

**TABLE 5:** Overview of multicenter comparison between LiCA and Cobas thyroid assays in serum specimens following the EP9‐A3 protocol

Assay	Institute	*N*	Sample range	Slope (95% CI)	Intercept (95% CI)	*R* (95% CI)	Median Bias (95% CI)	
							%	Value
TSH (mIU/L)	A	288	0.010 ~ 99.020	1.031 (1.009 ~ 1.052)	−0.063 (−0.097 ~ −0.039)	0.992* (0.990 ~ 0.994)	−3.55% (−7.01 ~ −1.10%)	−0.029 (−0.060 ~ −0.004)
	B	131	0.011 ~ 98.585	0.981 (0.958 ~ 0.995)	0.002 (−0.010 ~ 0.005)	0.995* (0.992 ~ 0.996)	−1.60% (−5.22 ~ 2.48%)	−0.015 (−0.076 ~ 0.004)
	C	122	0.010 ~ 99.533	0.998 (0.980 ~ 1.022)	−0.008 (−0.040 ~ 0.022)	0.994* (0.992 ~ 0.996)	−1.20% (−3.80 ~ 1.89%)	−0.008 (−0.068 ~ 0.029)
FT3 (pmol/L)	A	289	1.54 ~ 71.70	0.989 (0.962 ~ 1.013)	0.100 (−0.068 ~ 0.244)	0.981* (0.976 ~ 0.985)	0.40% (−2.07 ~ 2.17%)	0.02 (−0.14 ~ 0.14)
	B	140	1.54 ~ 73.74	1.045 (1.005 ~ 1.094)	−0.062 (−0.299 ~ 0.168)	0.977* (0.968 ~ 0.984)	3.15% (0.28 ~ 7.65%)	0.22 (0.02 ~ 0.48)
	C	126	2.05 ~ 69.82	1.012 (0.967 ~ 1.057)	−0.245 (−0.525 ~ 0.028)	0.966* (0.952 ~ 0.976)	−2.60% (−7.85 ~ 0.64%)	−0.16 (−0.41 ~ 0.06)
FT4 (pmol/L)	A	297	2.12 ~ 74.37	0.931 (0.896 ~ 0.966)	1.480 (0.913 ~ 2.010)	0.974* (0.967 ~ 0.979)	2.90% (0.33 ~ 5.69%)	0.47 (0.05 ~ 0.77)
	B	132	3.87 ~ 71.67	0.987 (0.951 ~ 1.024)	0.425 (−0.141 ~ 1.028)	0.983* (0.976 ~ 0.988)	1.40% (0.50 ~ 2.70%)	0.24 (0.06 ~ 0.50)
	C	128	3.86 ~ 74.39	0.946 (0.908 ~ 0.989)	0.345 (−0.405 ~ 1.138)	0.983* (0.975 ~ 0.988)	−3.60% (−5.10~−0.79%)	−0.58 (−0.96 ~ −0.18)
T3 (nmol/L)	A	255	0.26 ~ ~ 9.20	1.069 (1.025~1.115)	−0.193 (−0.305 ~ −0.093)	0.969* (0.961 ~ ~ 0.976)	−3.00% (−4.56 ~ 0.01%)	−0.06 (−0.10~0.01)
	B	128	0.62 ~ 8.97	0.915 (0.876 ~ 0.949)	0.278 (0.198 ~ −0.351)	0.965* (0.950 ~ 0.975)	3.95% (0.14 ~ 6.57%)	0.09 (0.01 ~ 0.14)
	C	124	0.62~9.15	0.992 (0.959 ~ 1.035)	0.092 (0.017 ~ −0.160)	0.974* (0.963 ~ 0.982)	4.35% (0.62 ~ 6.81%)	0.09 (0.02 ~ 0.12)
T4 (nmol/L)	A	254	7.87 ~ 360.06	0.946 (0.916 ~ 0.977)	1.744 (−2.032 ~ 3.967)	0.972* (0.964 ~ 0.978)	−3.95% (−6.20 ~ −2.20%)	−5.09 (−6.96 ~ −2.97)
	B	128	12.87 ~ 370.66	0.956 (0.909 ~ 1.001)	6.753 (2.458 ~ −10.688)	0.968* (0.955 ~ 0.977)	2.75% (0.59 ~ 6.41%)	2.50 (0.52 ~ 5.24)
	C	113	12.87 ~ 372.77	0.966 (0.898 ~ 1.042)	−0.924 (−9.289 ~ 5.250)	0.963* (0.947 ~ 0.975)	−4.70% (−8.82 ~ −1.82%)	−4.72 (−8.39 ~ −1.72)

Abbreviations: CI, confidence interval; *N*, sample number; *R*, Spearman's rank correlation coefficient.

*p < 0.0001.

### 3.6 Clinical performance

**FIGURE 2:**
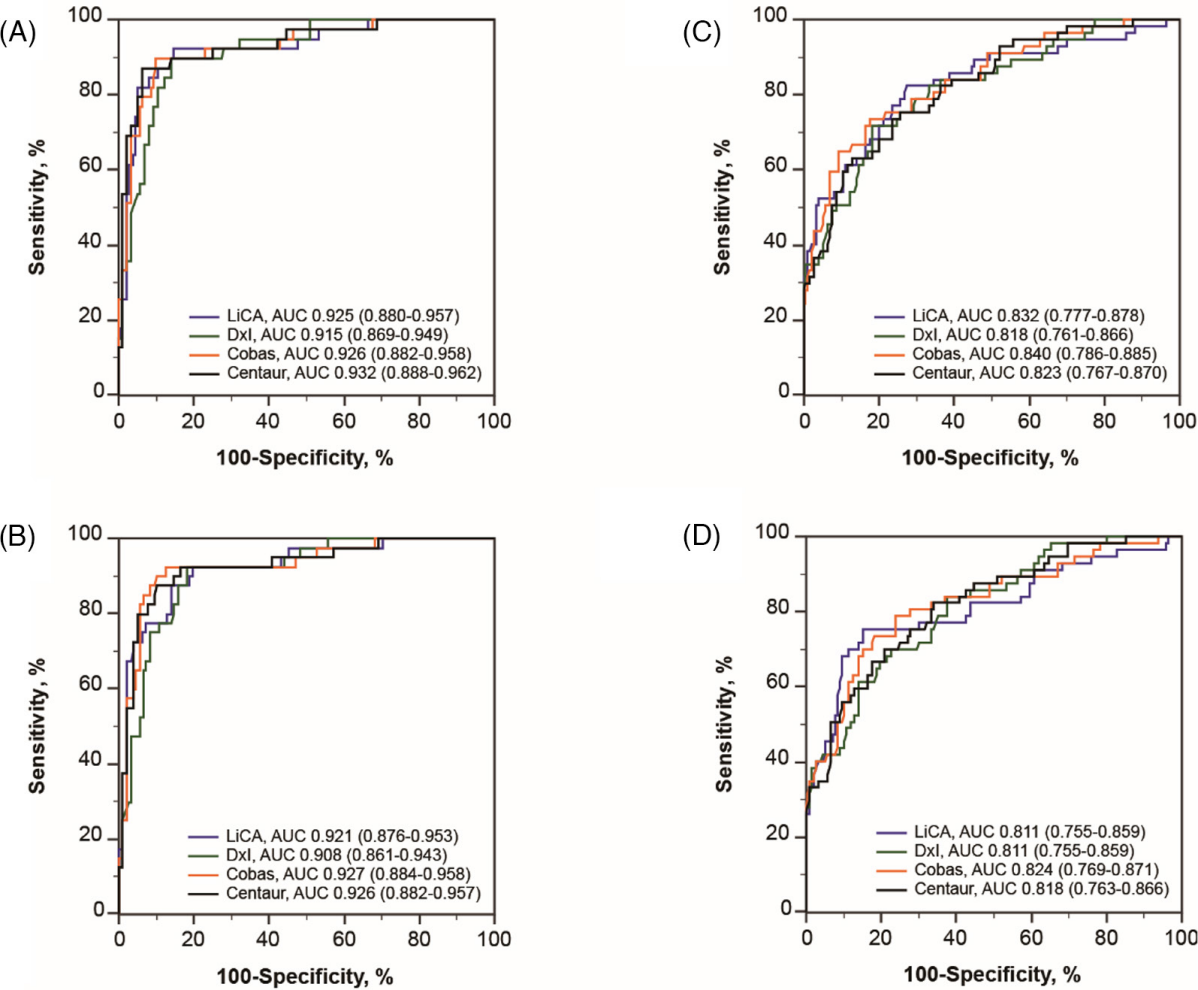
Receiver operating characteristic (ROC) curve analyses of thyroid assays for the diagnosis of hyperthyroidism and hypothyroidism. Abbreviations: AUC, area under curve

ROC curves were plotted in Figure 2 to describe the diagnostic accuracy of the assays. Using the five‐assay panel (TSH, FT3, FT4, T3, and T4) for the diagnosis of hyperthyroidism (Figure 2A) and hypothyroidism (Figure 2C), the AUCs (95% *CI*) were 0.925 (0.880–0.957) and 0.832 (0.777–0.878) for LiCA, comparable with DxI at 0.915 (0.869–0.949, *p *= 0.4678) and 0.818 (0.761–0.866, *p *= 0.6670), with Cobas at 0.926 (0.882–0.958, *p *= 0.8525) and 0.840 (0.786–0.885, *p *= 0.7955), with Centaur at 0.932 (0.888–0.962, *p *= 0.4283) and 0.823 (0.767–0.870, *p *= 0.7395), respectively. ROC curve analyses using the tree‐assay panel (TSH, FT3, and FT4) revealed a comparable diagnostic accuracy for hyperthyroidism (Figure 2B) and hypothyroidism (Figure 2D) between LiCA and DxI, Cobas, or Centaur, while the AUC was slightly lower than that generated by the five‐assay panel.

## 4 DISCUSSION

The essential principle of LiCA technology is singlet oxygen channeling through two kinds of nanoscale latex beads—sensitizer and emission.^7^, ^8^ The sensitizer contains the photosensitive dyes and is coated with streptavidin. The emission bead is coated with an assay‐specific antibody and contains the chemiluminescent dyes. Singlet oxygen transfer occurs only if the distance between two beads is within 200 nm (when the immune complex is formed) and triggers a cascade of chemical reactions. The reagent nanobeads provide three bases for the LiCA assays: (1) a fully homogeneous environment; (2) a larger specific surface area; and (3) a multi‐amplification signaling chemiluminescence. This enables LiCA offering a sensitive, no‐wash, rapid and stable immunoassay.^6^, ^7^, ^8^ Various assays based on this technology have been developed recently.^14^, ^15^, ^16^ In this study, we have validated the analytical and diagnostic performance of the LiCA thyroid assays.

Our data demonstrated a good precision for each of the five assays. Within‐run and within‐lab imprecisions across three levels of pooled sera were less than 6.5%. Satisfactory linearity was verified over the entire measuring range for TSH, T3, and T4 as previously claimed. Pearson's correlation coefficients were between 0.997–0.999 with all *p* values of 0.000, and the maximum change in recovery was within 8%. FT3 and FT4 are not applicable for linearity dilution, as there is an equilibrium for T3 and T4 in the blood with free and protein‐bound forms. A change in the concentration of binding proteins alters this equilibrium.

The analyzed LoD in this study agrees with the manufacturer's claim for each of the assays. However, there are no data issued for the LoQs in the product instructions for use. Our study may be one useful supplementary. Notably, the LoQ of LiCA TSH was determined to be 0.0019 mIU/L, and this was in accordance with the criteria of a fourth‐generation TSH method.^17^ A more extensive evaluation for LiCA TSH would be described in another work.

Paired‐comparison measurements demonstrated a comparable assay for each of the assays (*R* > 0.96, median bias <5%, *p *< 0.0001 all) between LiCA and Cobas across three institutes. To further characterize the clinical performance of the LiCA assays for the diagnosis of hyperthyroidism and hypothyroidism, we compared the AUCs with the five‐assay panel (TSH, FT3, FT4, T3, and T4) and with the three‐assay panel (TSH, FT3, and FT4) between LiCA and other three commonly used methods (DxI, Cobas, and Centaur). No significant difference was found (*p *> 0.3 all), and this confirmed the diagnostic accuracy and applicable in clinical practice of the LiCA thyroid assays.

In conclusion, LiCA 800 provides a precise and high‐throughput immunoassay platform for the detection of thyroid hormones. It is acceptable for clinical use.

## CONFLICT OF INTEREST

All authors state no conflict of interests.

## PATIENT CONSENT

All samples used in this study were collected from residual and de‐identified patient sera that were clinically tested for the thyroid assays. A waiver of the informed consent has been approved by the Biomedical Ethics Committee of Peking University International Hospital (No. 2021‐KY‐0002–01).

## PERMISSION TO REPRODUCE MATERIAL FROM OTHER SOURCES

Not applicable.

## CLINICAL TRIAL REGISTRATION

Not applicable.

## Data Availability

The datasets generated during the current study are available from the corresponding author on reasonable request.
